# Quantitative Trait Locus Analysis Implicates CD4^+^/CD44^high^ Memory T Cells in the Pathogenesis of Murine Autoimmune Pancreatitis

**DOI:** 10.1371/journal.pone.0136298

**Published:** 2015-09-01

**Authors:** Julia Bischof, Sarah Müller, Luise Borufka, Farahnaz Asghari, Steffen Möller, Stephanie-Anna Holzhüter, Horst Nizze, Saleh M. Ibrahim, Robert Jaster

**Affiliations:** 1 Department of Dermatology, University of Lübeck, Ratzeburger Allee 160, 23562 Lübeck, Germany; 2 Department of Medicine II, Division of Gastroenterology, Rostock University Medical Center, E.-Heydemann-Str. 6, 18057 Rostock, Germany; 3 Institute of Pathology, Rostock University Medical Center, Strempelstr. 14, 18057 Rostock, Germany; INSERM-Université Paris-Sud, FRANCE

## Abstract

The mouse strain MRL/MpJ is prone to spontaneously develop autoimmune pancreatitis (AIP). To elucidate the genetic control towards the development of the phenotype and to characterize contributions of immunocompetent cell types, MRL/MpJ mice were interbred with three additional strains (BXD2/TYJ, NZM2410/J, CAST/EIJ) for four generations in an advanced intercross line. Cellular phenotypes were determined by flow cytometric quantification of splenic leukocytes and complemented by the histopathological evaluation of pancreatic lesions. An Illumina SNP array was used for genotyping. QTL analyses were performed with the R implementation of *HAPPY*. Out of 41 leukocyte subpopulations (B cells, T cells and dendritic cells), only three were significantly associated with AIP: While CD4^+^/CD44^high^ memory T cells and CD4^+^/CD69^+^ T helper (Th) cells correlated positively with the disease, the cytotoxic T cell phenotype CD8^+^/CD44^low^ showed a negative correlation. A QTL for AIP on chromosome 2 overlapped with QTLs for CD4^+^/CD44^high^ and CD8^+^/CD44^high^ memory T cells, FoxP3^+^/CD4^+^ and FoxP3^+^/CD8^+^ regulatory T cells (Tregs), and CD8^+^/CD69^+^ cytotoxic T cells. On chromosome 6, overlapping QTLs for AIP and CD4^+^/IL17^+^ Th17 cells and again FoxP3^+^/CD8^+^ Tregs were observed. In conclusion, CD4^+^/CD44^high^ memory T cells are the only leukocyte subtype that could be linked to AIP both by correlation studies and from observed overlapping QTL. The potential role of this cell type in the pathogenesis of AIP warrants further investigations.

## Introduction

Autoimmune pancreatitis (AIP) represents a rare form of chronic pancreatitis that is nevertheless clinically important for two reasons: First, AIP is a differential diagnosis of pancreatic cancer, a devastating disease where radical surgery as the only curative therapy is applicable in a minority of patients only. Secondly, therapy of AIP differs not only from the therapy of pancreatic cancer, but also from the treatment of other, more common forms of chronic pancreatitis: AIP is highly sensitive to immunosuppressants and can usually be successfully treated with steroids (reviewed in [1]). Hence, there is need for an early and reliable diagnostics of AIP, which remains challenging despite the availability of diagnostic algorithms that consider radiological, serological and histological criteria [2].

To a large extent, improvement of AIP diagnostics (and therapy) is currently limited by the incomplete knowledge of the pathogenesis of the disease. The initial triggering mechanisms are largely unknown, and the specific contribution of individual autoantigens (mainly digestive enzymes of pancreatic acinar cells, but also proteins from pancreatic duct cells [1,3,4]) is under debate.

Recent concepts distinguish between two subtypes of AIP. The pathognomonic finding in AIP of subtype 1 is the abundant infiltration of pancreatic tissue with lymphocytes and IgG4^+^ plasma cells. This form of AIP is now considered as the pancreatic manifestation of a multi-organ disease, which has recently been named IgG4-related disease. In AIP subtype 2, the typical histopathologic pattern includes granulocytic epithelial lesions, while IgG4^+^ plasma cells are lacking [5,6]. Both forms of AIP are characterized by a progressive replacement of pancreatic tissue by connective tissue (fibrosis), formation of inflammatory pseudotumors, and clinically by frequent presentation with obstructive jaundice [1].

AIP shares with other autoimmune diseases key features such as genetic predisposition (outlined below), detectability of autoantibodies and the presence of autoreactive T cells [1]. The observation that depletion of B cells with anti-CD20 antibodies is an effective therapy for AIP [7] has demonstrated a crucial involvement of these immune cells in the pathogenesis of the disease. In addition, a growing body of evidence implicates different subsets of T cells in the progression of AIP. Thus, both T helper (Th) 1 and Th2 immune responses have been described to occur in AIP [1,8–10], and the possibility that the Th1/Th2 balance may shift dynamically between early and advanced AIP has been suggested [1]. Own studies in MRL/MpJ mice, a model of spontaneous AIP [11], have shown a key role of effector T cells in the development of the disease, but also implicated regulatory T cells (Tregs) in the suppression of AIP progression [12,13]. In AIP patients, increased numbers of Tregs have been detected both in pancreatic tissue [10] and peripheral blood [14]. Together, these observations suggest a complex interplay between components of cellular and humoral autoimmunity in AIP, the details of which still remain to be elucidated.

Multifactorial diseases, such as autoimmune and chronic inflammatory disorders, are considered to be influenced both by environmental and genetic factors [15–18]. With respect to the genetic basis of AIP, genome-wide association studies are hampered by the lack of large cohorts of patients. Nevertheless, some genetic risk factors of human AIP have already been proposed: In a Japanese population, the HLA serotypes DRB1*0405 and DQB1*0401 were found to increase the susceptibility to AIP [19]. In patients from Korea, a mutation of DQbeta1 (substitution of aspartic acid at position 57) was identified as a key genetic factor for relapse of AIP [20]. Furthermore, single-nucleotide polymorphisms (SNPs) in five non-HLA genes, *cytotoxic T lymphocyte-associated antigen 4* (*CTLA4*) [21,22], *tumor necrosis factor-α* (*TNF-α*) [22], *cationic trypsinogen* (*PRSS1*) [23], *Fc receptor-like 3* (*FCRL3*) [24], and *KCNA3* (*potassium voltage-gated channel*, *shaker-related subfamily*) [25] have been linked to AIP.

Given the problems of obtaining large sizes of human samples, studies in animal models of AIP may provide valuable additional insights into the genetic basis of the disease. We have previously established an advanced intercross line originating from MRL/MpJ parental mice and three other mouse strains: CAST/EIJ (healthy controls), BXD2/TYJ (susceptible to collagen-induced arthritis) and NZM2410/J (a model of Lupus erythematodes) [26]. The idea beyond this concept was to map both general autoimmune disease-associated loci and AIP-specific quantitative traits. Therefore, generation 4 of outbread intercross mice was characterized phenotypically by scoring histopathological changes of the pancreas and genotyped employing SNP arrays. By this approach, five quantitative trait loci (QTL), located on chromosomes 2, 4 (n = 2), 5 and 6, were mapped [26]. Similarly, we identified QTLs controlling arthritis and skin inflammation [27,28]. Taking sex as a covariate, we have now used the same approach to study genomic loci that control immune cell phenotypes in the spleen and to determine their overlap with the QTLs for AIP. Out of several leukocyte subtypes, only CD4^+^/CD44^high^ memory T cells where *not only* controlled by such an overlapping QTL, but also showed a significant correlation of their relative frequency with the appearance of AIP.

## Materials and Methods

### Animal Model and Experimental AIP

The establishment of the 4-way autoimmunity advanced intercross line has been described before [26]. Briefly, MRL/MpJ, NZM2410/J, BXD2/TyJ and CAST/EiJ parental mouse strains were intercrossed at an equal strain and sex distribution. To maintain an equal distribution of original strains in subsequent generations, parental origin of offspring mice of the predecessor generation was considered. For each generation of mice, at least 50 breeding pairs were used as parentals. As previously described, MRL/MpJ mice, but no individuals of the other parental strains, developed AIP in an age and gender specific manner [26,29].

Development of spontaneous AIP in parental strains and in intercross generation 4 (156 males and 175 females) was assessed in 6-months-old mice by evaluating the severity of pancreatic lesions. Therefore, paraffin-embedded pancreatic sections were stained with hematoxylin and eosin (H&E), applying standard protocols. Pathological changes were graded on a semi-quantitative scale from 0 to 4 [26]. The stages were defined as follows: 0, no pathological changes; 1, minimal infiltration of periductal tissue with mononuclear cells but no parenchymal destruction; 2, moderate periductal infiltration with mononuclear cells associated with beginning parenchymal destruction; 3, severe periductal inflammation and/or more extended parenchymal destruction; 4, diffuse mononuclear cell infiltrates, destruction of acini and (partial) replacement by adipose tissue. All samples were assessed by two independent investigators and blinded before evaluation. AIP stages were determined by microscopic analysis of at least two tissue sections per sample. Mice with pancreatic lesions that scored ≥ 2 were defined as positive for AIP.

Animals were kept under specific pathogen-free conditions at a 12 h light/dark cycle with food and water ad libitum. All procedures were performed with adherence to the EU Directive 2010/63/EU for animal experiments and approved by the local governmental administrations (Landesamt für Landwirtschaft, Lebensmittelsicherheit und Fischerei Mecklenburg-Vorpommern).

### Immunohistochemical Analysis

Cryostat sections of pancreatic tissue (6 μm) were fixed by incubation in ice-cold methanol for 1 min at 4°C and washed three times with PBS. Subsequently, they were stained using the Vectastain ABC staining kit (Vector Laboratories, Burlingame, CA, USA) according to the manufacturer’s instructions. For the detection of CD4 and CD44, mouse-specific primary rat antibodies were employed (anti-CD4, Immunotools, Friesoythe, Germany and anti-CD44, eBioscience, San Diego, CA, USA, respectively). The sections were counterstained with hemalaun and examined by light microscopy (Axioskop 40, Zeiss, Oberkochen, Germany).

### Analysis of Leukocyte Subtypes by Flow Cytometry

Splenocytes were isolated from the spleen of G4 mice using a cell strainer (70 μm). Red blood cells were lysed applying RBC lysis buffer (eBioscience) according to the manufacturer’s instructions. After washing and centrifugation steps, 1x10^6^ cells per stain were subjected to subsequent analysis. Prior to staining, Fc receptors on splenocytes were blocked by pre-incubation with anti-CD16/CD32 antibodies (BD Biosciences, Heidelberg, Germany) for 5–10 minutes on ice. Surface staining was performed by incubating the cells with fluorochrome-conjugated specific antibodies (listed in the supplement, S1 Table) for at least 20 min in dark on ice. After washing and centrifugation steps, stained cells were fixed with 1% paraformaldehyde for 10–20 min at 4°C and subjected to flow cytometry.

For staining of intracellular cytokines, single cell suspensions of splenocytes were fixed in 4% paraformaldehyde for 10–20 min at 4°C and permeabilized employing Saponin (0.3%; Sigma-Aldrich, Deisenhofen, Germany) for 10 min. Afterwards, optimized concentrations of fluorochrome-conjugated anti-cytokine antibodies were applied at 4°C for 30 min in the dark, followed by washing steps and flow cytometry.

FACS analysis was performed using a FACSCalibur cytometer (BD Biosciences). 10,000 events were measured for each sample, and the data were processed employing the BD CellQuest software. In total, 41 different subsets of leukocytes were analyzed. Investigations included B cells (n = 6 phenotypes); cytotoxic T cells (n = 8 subtypes); CD4/CD8 double-positive T cells; Th1, Th2 and Th17 lymphocytes as well as otherwise defined subsets of Th cells (n = 10); Tregs (n = 2); further phenotypes of T cells (n = 4); conventional dendritic cells (conventional DCs; n = 3) and plasmacytoid DCs (PD; n = 4). With the following exceptions, all FACS antibodies were obtained from BD Biosciences: anti-CD4 and anti-CD45R (B220)—Immunotools (Friesoythe, Germany), anti-MHC class II, anti-PDCA-1 and anti-FoxP3 –eBioscience, and anti-CD8 –Santa Cruz Biotechnologies (Santa Cruz, CA, USA).

Details regarding marker combinations and the corresponding leukocyte subpopulations are given in Table 1. The table also indicates the averaged results for each phenotype, obtained from the analysis of 331 G4 mice. Data are expressed as relative frequency of the investigated leukocyte subset, with 100% corresponding to the total number of splenocytes. Representative dot plots of the experimental data are shown in the supplement (S1 Fig).

**Table 1:** Summary of immune cell phenotypes of mice splenocytes detected by flow cytometry. Results are expressed as percentage (mean ± SD) of total splenocytes from 331 G4 mice. MHC II: major histocompatibility complex class II, FoxP3: forkhead box protein P3, IFNγ: interferon-γ, IL4: interleukin-4, IL17: interleukin-17, PDCA1: plasmacytoid dendritic cell antigen-1. Cell types were defined as follows: B cells–CD19^+^, T cells–CD3^+^, double-positive (DP) T cells–CD4^+^/CD8^+^, cytotoxic T cells–CD8^+^, T helper cells–CD4^+^, Th1 cells–CD4^+^/IFNγ^+^, Th2 cells—CD4^+^/IL4^+^, Th17 cells—CD4^+^/IL17^+^, Tregs–FoxP3^+^, conventional dendritic cells (cDCs)–CD11c^+^, Plasmacytoid dendritic cells (PD)–PDAC1^+^.

Cell Type			Marker	Mean ± SD
**B cells**			CD19^+^/CD69^-^	36.81 ± 11.13
			CD19^+^/CD69^+^	0.29 ± 0.21
			CD19^+^/CD86^-^	38.65 ± 9.78
			CD19^+^/CD86^+^	0.41 ± 0.36
			CD19^+^/ MHC II^-^	5.15 ± 5.7
			CD19^+^/ MHC II^+^	32.51 ± 11.6
**T cells**			CD3^+^/CD8^+^	8.3 ± 4.06
			CD8^+^/CD44^low^	4.2 ± 3.12
			CD8^+^/CD44^high^	4.6 ± 3.47
	cytotoxic		CD8^+^/CD62L^-^	2.5 ± 2.38
	T cells		CD8^+^/CD62L^+^	6.07 ± 3.81
			CD8^+^/CD69^-^	8 ± 4.59
			CD8^+^/CD69^+^	0.38 ± 0.37
			CD8^+^/FoxP3^-^	8.36 ± 4.56
	DP T cells		CD4^+^/CD8^+^	0.57 ± 0.38
		Th 1	CD4^+^/IFNγ^+^	0.48 ± 0.6
		Th 2	CD4^+^/IL4^+^	0.21 ± 0.21
		Th 17	CD4^+^/IL17^+^	0.29 ± 0.27
			CD3^+^/CD4^+^	15.5 ± 5.59
			CD4^+^/CD8^-^	15.66 ± 5.6
	T helper		CD4^+^/CD69^-^	16.4 ± 5.33
	cells		CD4^+^/CD69^+^	0.33 ± 0.68
		others	CD4^+^/CD44^low^	4.54 ± 3.44
			CD4^+^/CD44^high^	12.63 ± 4.34
			CD4^+^/CD62L^-^	9.39 ± 3.48
			CD4^+^/CD62L^+^	7.54 ± 4.65
			CD4^+^/FoxP3^-^	17.64 ± 6.93
			CD4^+^/IFNγ^-^	17.58 ± 6.27
	Tregs		CD4^+^/FoxP3^+^	0.41 ± 0.38
			CD8^+^/FoxP3^+^	0.14 ± 0.14
			CD3^+^/CD4^-^	9.81 ± 4.13
	others		CD3^+^/CD8^-^	17.01 ± 5.75
			CD3^+^/B220^-^	22.91 ± 8.6
			CD3^+^/B220^+^	2.32 ± 1.01
**DCs**			PDCA1^+^/CD86^-^	3.51 ± 3.52
	PD cells		PDCA1^+^/CD86^+^	0.46 ± 0.54
			PDCA1^+^/CD11c^-^	2.33 ± 2.4
			PDCA1^+^/CD11c^+^	0.76 ± 0.46
			CD11c^+^/CD86^-^	2.43 ± 1.21
	cDCs		CD11c^+^/CD86^+^	0.32 ± 0.32
			CD11c^+^/PDCA1^-^	1.95 ± 1.01

### Correlation Analysis

Correlations between AIP appearance and sex, as well as between AIP and leukocyte subpopulations, were analyzed using Spearman’s correlation coefficient (*Spearman’s rho*). Significance of correlations was calculated using a correlation test in R. Correction for multiple testing was done by controlling the false discovery rate using the Benjamini-Hochberg approach.

### QTL Mapping

331 G4 mice were genotyped by a set of 1400 single nucleotide polymorphisms (SNPs) using an Illumina murine HD array (Illumina, California, USA). To provide QTL coordinates, NCBI Build 36 was used. Genotype/phenotype correlation was performed with the R package of *HAPPY* [30] in version 2.3 beta on Debian Linux [31]. The default parameters were applied, including four generations. For the analysis of the AIP phenotype binomial stages (AIP stages <2 and ≥2) were used and for the immune phenotypes relative frequencies. Using sex as covariate, the data of all chromosomes were analyzed together in an additive model, and tested positively against 1000 permutations for global significance (p<0.05). The flanking regions of QTLs were determined manually by a drop of 1.5 of the-log p value to both sides of the peak.

## Results

### Pancreatic Histopathology and Immune Cell Phenotypes of G4 Mice

**Fig 1:**
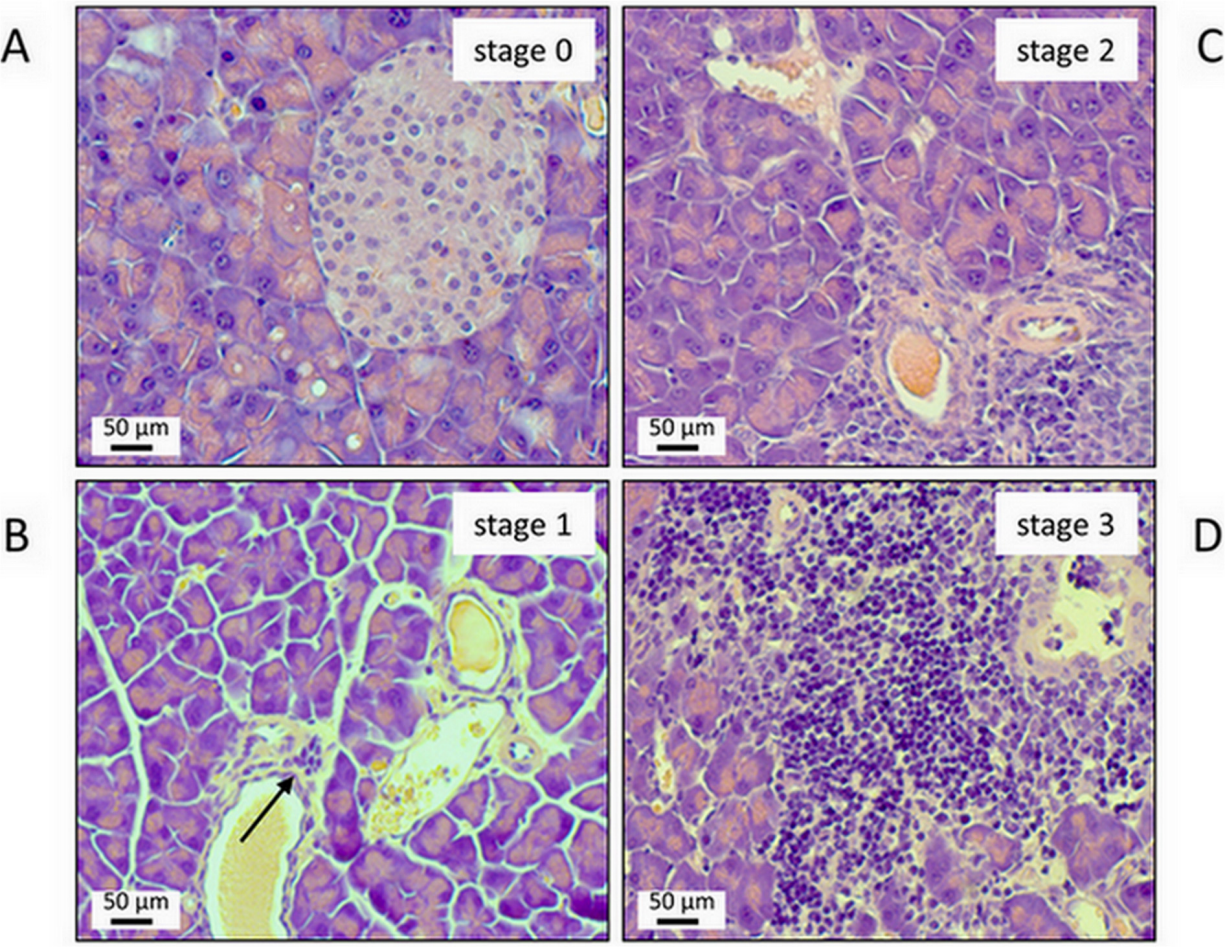
Representative examples of pancreatic lesions ranging from stage 0 (A) to 3 (D). Sections of paraffin-embedded pancreatic tissue were stained with H&E. (A) healthy pancreas; stage 0. (B) minimal lymphocytic infiltration of the subepithelial layer of one larger duct (arrow); no parenchymal destruction; stage 1. (C) more extended lymphocytic infiltration; beginning destruction of acinar tissue; stage 2. (D) severe periductal inflammation with progressive parenchymal destruction; stage 3.

Generation 4 of the 4-way autoimmune-prone intercross mouse line was employed, at an age of 6 months, to assess pancreatic histopathology and relative frequencies of different leukocyte subsets in the spleen. Therefore, 331 mice (156 females and 175 males) were included into the investigations. The mice represent a subset of the previously analyzed 351 animals [26], which was chosen based on the availability of flow cytometry data. Scoring of AIP-typical pancreatic lesions, such as presence of lymphocytic foci and parenchymal destruction, revealed an AIP stage 2 or 3 in 44 mice (32 females and 12 males). Stage 4 was not detected, but has been observed in mice of the advanced intercross line outside of this study (R.J., unpublished data). These numbers correspond to 18.3% of the females but only 7.7% of the males (13.3% of all mice). Details are given in Table 2. Exemplary tissue stains are shown in Fig 1.

**Table 2:** Pancreatic histopathology. Pancreatic sections of 331 G4 mice were stained with H&E, and evaluated applying a scoring system from 0 to 4 as further described in the methods section. The data are a subset of previously published data obtained from 351 mice [26].

Gender	Number	AIP stage				AIP stage
		0	1	2	3	2 + 3 (%)
Female	175	49	94	31	1	18.3
Male	156	77	67	11	1	7.7
Both	331	126	161	42	2	13.3

The data are in line with our previous report [26], indicating that the 331 mice are representative of the entire cohort. They are also in agreement with the fact that AIP of the parental MRL/MpJ strain is largely restricted to females (at an age of at least 6 months) [11,29]. Gender dependency of AIP was also confirmed by correlation analysis (*Spearman's rho* = -0.16; p = 0.0090).

To study immune cell phenotypes in the context of AIP, splenocytes were phenotyped employing flow cytometry. We focused on 41 subsets of leukocytes (B cells, T cells and DCs), covering a large range of immunocompetent cells that have been implicated in the pathogenesis of various autoimmune diseases [32,33]. Special attention was paid to B cells, cytotoxic and helper T cells, Tregs and DCs. Table 1 in the *Materials and methods* section shows the relative frequencies of all these leukocyte subtypes as averaged values of the 331 G4 mice.

### Correlations between AIP and Leukocyte Abundance

We next studied correlations between appearance of AIP and the relative frequencies of the 41 leukocyte subsets (Table 3).

**Table 3:** Significant correlations between AIP incidence and lymphocyte cell types, using Spearman’s correlation coefficient. P values are from correlation tests. Correction for multiple testing was performed using the Benjamini-Hochberg approach. A corrected p value of p<0.05 (indicated in bold) was considered statistically significant.

Subpopulation	Cell type	Spearman'srho	p value	Corrected p value
cytotoxic T cells	CD8^+^/CD44^low^	-0.16	0.0023	**<0.05**
cytotoxic T cells	CD8^+^/CD69^+^	0.10	0.0498	>0.05
T helper cells	CD4^+^/CD44^low^	-0.15	0.0053	>0.05
T helper cells	CD4^+^/CD62L^-^	0.12	0.0245	>0.05
T helper cells	CD4^+^/CD69^+^	0.16	0.0030	**<0.05**
T helper cells	CD4^+^/CD44^high^	0.19	0.0003	**<0.05**

A significant correlation (corrected p<0.05) was found for three populations of lymphocytes: activated Th cells (CD4^+^/CD69^+^), CD4^+^/CD44^high^ memory T cells, and cytotoxic T cells of the phenotype CD8^+^/CD44^low^. While the first two leukocyte subsets were positively correlated with the disease, CD8^+^/CD44^low^ cells displayed a negative correlation. For three further lymphocyte populations, p values <0.05 were determined only prior to correction for multiple testing. These cell types are activated cytotoxic T cells (CD8^+^/CD69^+^; positive correlation), and Th cells with the phenotypes CD4^+^/CD62L^-^ (positive correlation) and CD4^+^/CD44^low^ (negative correlation).

### QTL Analysis

We have previously employed Illumina murine HD arrays to genotype 351 G4 mice of the 4-way autoimmune-prone intercross mouse line [26]. Here, we have re-analyzed the data subset of the 331 phenotyped mice (see above) to perform QTL analyses with sex as covariate. As shown in Table 4, a total of four QTLs could be mapped to the chromosomes 2, 4 and 6 (global p<0.05). The–log10(p) values varied from 4.2 to 5.5. To avoid any confusion with the previously identified QTLs, these new QTLs were termed AIP s1-s4. The mean confidence interval (CI) of each QTL was roughly 19 Mb.

**Table 4:** Significant QTLs found for AIP (1000 permutations; global p value <0.05). QTLs are listed with chromosome, peak position and confidence interval (CI). –log10(p) belongs to marker at peak position.

QTL	Chromo-some	Peak position (Mbp)	CI (Mbp)	-log10(p)
AIP s1	2	59.9	56.4–73.3	4.23
AIP s2	4	82.8	79.3–101.1	4.61
AIP s3	6	104.8	94–106.3	4.31
AIP s4	6	116.7	109.9–136.3	5.54

Next, the same genotyping data subset was used to determine QTLs of all 41 leukocyte subpopulations (Table 5; S2 Table). Most significant QTLs (global p<0.05) were found for B cells (n = 80), T helper cells (n = 68) and cytotoxic T cells (n = 59). For conventional and plasmacytoid DCs, 8 and 7 QTLs, respectively, could be mapped, while for Tregs 25 loci were detected. Only 4 leukocyte subsets (CD19^+^/CD86^+^, CD8^+^/CD62L^-^, CD4^+^/CD69^+^ and PDCA1^+^/CD11c^-^ cells) were not influenced by any QTL, whereas for CD19^+^/CD69^-^, CD19^+^/MHC II^-^ and CD8^+^/CD69^+^ lymphocytes more than 20 QTLs were discovered. The mean CI of each QTL was about 13.6 Mb. -log10(p) values varied from 3.8 to 32.5 (S2 Table). QTLs were located on all chromosomes, but most were found on chromosomes 2, 6, 7, 9, 12 and 20 (Table 5). For B cells, most QTLs were mapped on chromosomes 8 and 9, for cytotoxic T cells on chromosome 20 and for T helper cells on chromosome 17 (Table 5).

**Table 5:** Number of significant QTLs found for leukocyte cell types (1000 permutations; global p value <0.05) on each chromosome. For abbreviations and definition of cell types, please refer to Table 1.

Cell Type			Marker	Chromosome																			
				1	2	3	4	5	6	7	8	9	10	11	12	13	14	15	16	17	18	19	20
**B cells**			CD19^+^/CD69^-^		2	3	1	3	4	4	3	3							1				2
			CD19^+^/CD69^+^	2	2						2						6						
			CD19^+^/CD86^-^					3	2	1		3							1				2
			CD19^+^/CD86^+^																				
			CD19^+^/ MHC II^-^		2	1	1													1			
			CD19^+^/ MHC II^+^		2	2		2	2	2	4	3		1				1	1	1	2		2
**T cells**			CD3^+^/CD8^+^	1											3								3
			CD8^+^/CD44^low^		1		2					1			4								3
			CD8^+^/CD44^high^		2																		
	cytotoxic		CD8^+^/CD62L^-^																					
	T cells		CD8^+^/CD62L^+^			1	1					1			1								3
			CD8^+^/CD69^-^																				1
			CD8^+^/CD69^+^	2	4	2	3	3	2	2	1			2			1			1	1	2	
			CD8^+^/FoxP3^-^							1					1		1						2
	DP T cells		CD4^+^/CD8^+^			1				1						1							1
		Th 1	CD4^+^/IFNγ^+^					2				1	1	1	1			1			3	1		
		Th 2	CD4^+^/IL4^+^		1		1				1			1									
		Th 17	CD4^+^/IL17^+^	1			2		1			1											2	
			CD3^+^/CD4^+^						1	1					2					1			
			CD4^+^/CD8^-^	1					1						2					1			
	T helper		CD4^+^/CD69^-^						1						2					1			1
	cells		CD4^+^/CD69^+^																				
		others	CD4^+^/CD44^low^		1		1				1									1			
			CD4^+^/CD44^high^		1				3							1		1				2	
			CD4^+^/CD62L^-^		2	2									1								
			CD4^+^/CD62L^+^		2			1						1						2			
			CD4^+^/FoxP3^-^			1				1				1						1			
			CD4^+^/IFNγ^-^	1						1												2	
	Tregs		CD4^+^/FoxP3^+^				4			2		1		1		2		1	1	1		1	1
			CD8^+^/FoxP3^+^		3				2	2		2						1					
			CD3^+^/CD4^-^	1					1						2								2
	others		CD3^+^/CD8^-^						1	1					2								
			CD3^+^/B220^-^												2								1
			CD3^+^/B220^+^		2		1					1	1						1				
**DCs**			PDCA1^+^/CD86^-^														1		1		1		
	PD cells		PDCA1^+^/CD86^+^										1										
			PDCA1^+^/CD11c^-^																				
			PDCA1^+^/CD11c^+^									3											
			CD11c^+^/CD86^-^												1						1		
	cDCs		CD11c^+^/CD86^+^										1										
			CD11c^+^/PDCA1^-^	1						2											2		

### Overlapping QTLs for Immune Cell Phenotypes and AIP

The significant QTLs for AIP with sex as a covariate are located on chromosomes 2, 4 and 6 (Table 4). Of these, chromosomes 2 and 6 also harbor significant QTLs for B cells, cytotoxic T cells, T helper cells and Tregs. On chromosome 4, we identified QTLs for the same subpopulations, except of Tregs (Table 5). No QTLs for conventional or plasmacytoid DCs are located on these disease-associated chromosomes.

**Fig 2:**
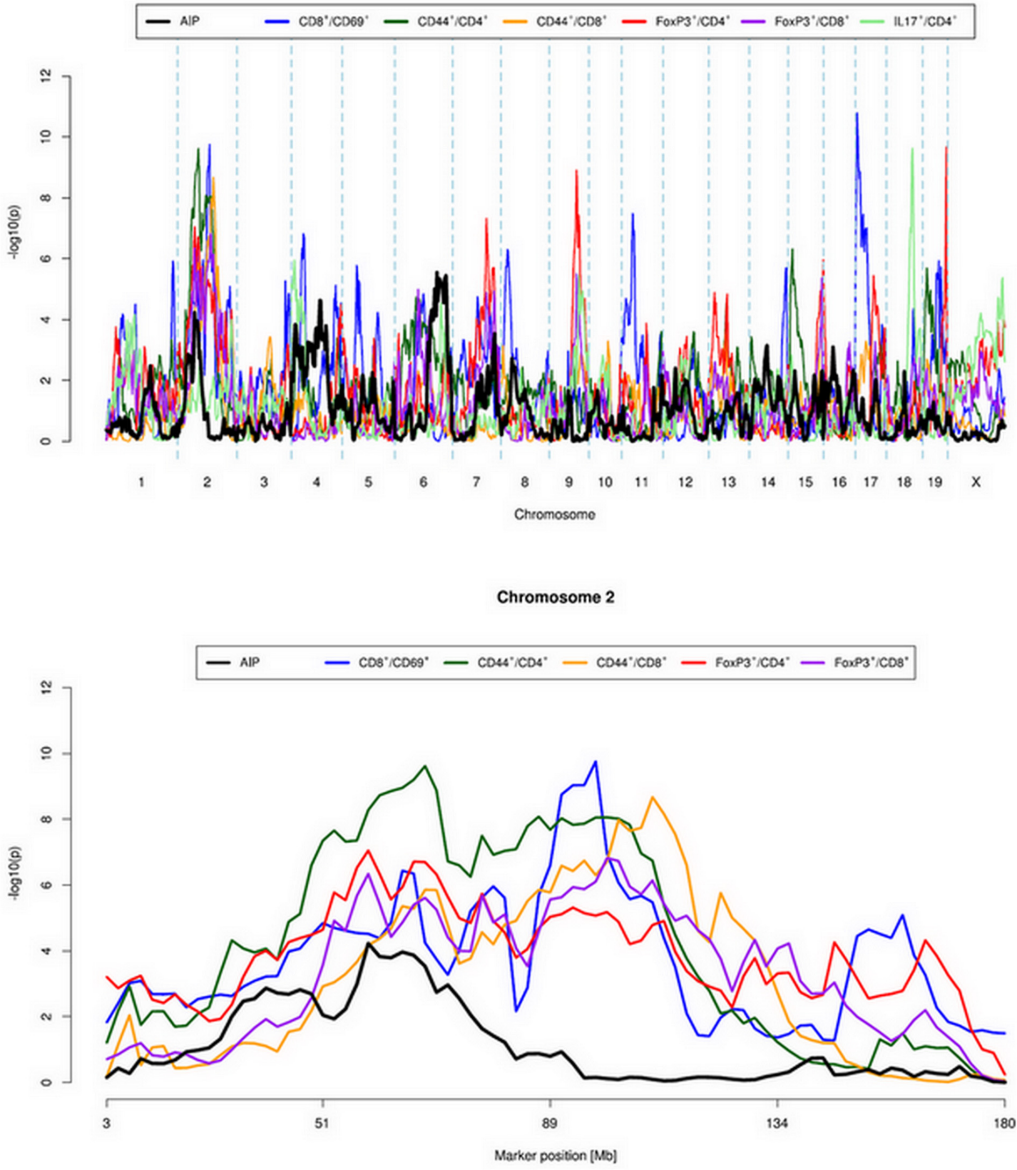
Quantitative trait loci (QTLs) for autoimmune pancreatitis and leukocyte subsets. The figure shows plots of QTLs for autoimmune pancreatitis and for leukocyte subsets with overlapping QTLs (upper panel: full genome; lower panel: chromosome 2). Log-likelihood values were determined using *HAPPY*. Logarithmic p values are presented in relation to positions of the marker loci along the chromosome. For confidence intervals and overlapping QTLs on chromosome 6, please refer to Table 6.

In subsequent analyses, QTL were considered as overlapping when their peaks are no more than 10 Mb apart. Interestingly, for the QTLs AIP s1, AIP s3 and AIP s4 such overlaps with QTLs for individual leukocyte subsets could be observed (Fig 2, Table 6). The peak of QTL AIP s1 on chromosome 2 was next to peaks of QTLs for subtypes of T helper cells (CD4^+^/CD44^high^), Tregs (FoxP3^+^/CD4^+^, FoxP3^+^/CD8^+^) and cytotoxic T cells (CD8^+^/CD69^+^, CD8^+^/CD44^high^). On chromosome 6, the QTLs AIP s3 and AIP s4 overlapped with QTLs for Tregs (again, FoxP3^+^/CD8^+^) and Th17 cells (IL17^+^/CD4^+^), respectively.

**Table 6:** Overlapping QTLs for AIP and leukocyte subtypes with distances between peaks below 10 Mbp. QTLs are listed with chromosome (Chr), peak position, confidence interval (CI) and –log10(p).

Cells/QTL (bold)	Phenotype	Chr	Peak (Mbp)	CI (Mbp)	-log10(p)
**AIP s1**	AIP	2	59.9	56.4–73.3	4.23
T regulatory cells	FoxP3^+^/CD4^+^	2	59.9	56.4–73.3	7.05
T regulatory cells	FoxP3^+^/CD8^+^	2	59.9	56.4–61.6	6.34
Cytotoxic T cells	CD8^+^/CD69^+^	2	65.0	65–66.8	6.43
T helper cells	CD44^high^/CD4^+^	2	68.1	59.9–71.4	9.62
Cytotoxic T cells	CD44^high^/CD8^+^	2	68.1	61.6–73.3	5.86
**AIP s3**	AIP	6	104.8	94–106.3	4.31
T regulatory cells	FoxP3^+^/CD8^+^	6	97.9	84.8–98.4	4.12
**AIP s4**	AIP	6	116.7	109.9–136.3	5.54
Th17 cells	IL17^+^/CD4^+^	6	125.0	125–130.2	4.54

### Detection of CD44 on Lymphocytes in Pancreatic Tissue

**Fig 3:**
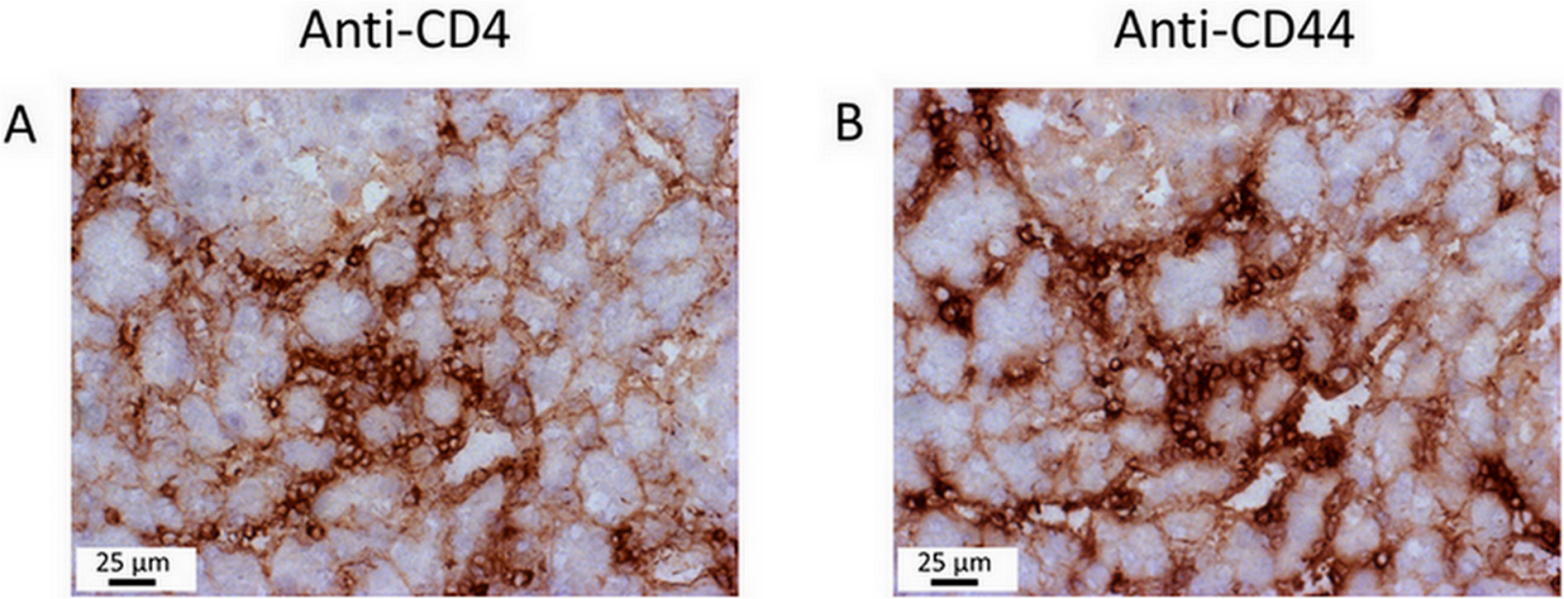
Immunohistochemical analysis of leukocyte infiltrates in the mouse pancreas. Serial pancreatic sections from a mouse with AIP stage 2 were stained with the ABC staining kit, using primary antibodies against (A) CD4 and (B) CD44. Note the pronounced cellular infiltration within the focal inflammatory lesions. Analysis of further mice, with AIP stages between 1 and 3, yielded similar results.

CD4^+^/CD44^high^ lymphocytes were the only leukocyte subtype that could be linked to AIP both by the correlation studies (Table 3) and from observed overlapping QTL (Table 6). We therefore asked if cells with this phenotype were also present in the inflamed pancreatic tissue. Using serial sections, we found that cells expressing CD4 and CD44, respectively, could be detected in overlapping regions of lymphocytic foci (Fig 3).

## Discussion

As other autoimmune diseases, AIP is considered to develop after environmental triggering of genetically susceptible individuals [1,15–18,32,33]. Except of a few HLA serotypes and SNP polymorphisms in non-HLA genes, however, no AIP-associated genetic factors have been identified in humans so far. This is largely due to the relative rarity of the disease (estimated prevalence in the general population: <1/100,000), which hampers the buildup of large cohorts of patients.

Employing an autoimmune-prone intercross mouse line, we previously identified five QTLs of murine AIP that may provide additional insights into the pathogenesis of the disease [26]. In our current work, we expanded these investigations by analyzing AIP-associated immune cell phenotypes and their genetic control. Since AIP of the parental MRL/MpJ mouse strain is more frequent in females, sex was considered as a covariate in all our investigations. Taking this approach, we confirmed the susceptibility loci on mouse chromosomes 2 (CI 56.3–81.9 Mb), 4 (CI 81.3–120.7 Mb) and 6 (CI 111.2–133.9 Mb) (Table 4). Since our previous analysis [26] was performed *without* sex as covariate, these QTLs can now be assigned as sex-independent. Neither here nor in our previous study, we could identify any X-linked QTL, suggesting that the effect of X-linked genes is too small to be verified by our approach. The reason for the higher susceptibility of the female mice to AIP, therefore, remains to be deciphered.

Recently, Okada *et al*. reported a pathogenetic role of *mag*, an autoimmune susceptibility locus encoded by the telomeric region of MRL/MpJ mouse chromosome 1, in murine AIP [34]. Interestingly, this susceptibility locus is not preserved in our intercross line since we did not map significant genetic markers on chromosome 1.

In a comprehensive effort, we phenotyped a variety of B cell, T cell and dendritic cell subtypes by determining their relative frequencies in the spleen of G4 mice, and systematically searched for genetic loci that influence these phenotypes. We came up with no less than 273 QTLs that control 37 different splenocyte subtypes (Table 5). This original set of loci was considerably narrowed down when an overlap with one of the AIP QTLs was introduced as an additional criterion: Of the immune cell-associated QTLs, only five overlapped with the QTL AIP s1, and one each with AIP s3 and AIP s4 (Table 6). The corresponding immune cell phenotypes consist of activated cytotoxic T cells (CD8^+^/CD69^+^), Th17 cells (CD4^+^/IL17^+^), two types of T cells expressing the memory marker CD44 (CD4^+^/CD44^high^ and CD8^+^/CD44^high^), and also two types of regulatory T cells (FoxP3^+^/CD4^+^ and FoxP3^+^/CD8^+^; with the latter being influenced by two distinct loci).

So far, we had identified a small subset of QTLs that influence both the appearance of AIP *and* control a total of six different immune cell phenotypes in the spleen. These data, however, did not address the question of a direct association between the two phenotypes (AIP and frequency of the respective immune cells). Therefore, a correlation analysis (Table 2) was performed which revealed a statistically significant *negative* correlation between AIP and cytotoxic T cells of the phenotype CD8^+^/CD44^low^, and a *positive* such for AIP and activated T helper cells (CD4^+^/CD69^+^) as well as CD4^+^/CD44^high^ cells. For the first two T cell subtypes, no QTLs overlapping with QTLs for AIP had been found. CD4^+^ cells expressing the memory marker CD44 are therefore the only cell type which could be linked to AIP by both approaches. Both cell surface markers could also be detected on immune cells in pancreatic lesions (Fig 3). The latter findings complement our previous studies regarding the composition of the immune cell infiltrate in MRL/MpJ mice, which had shown a predominance of CD4 cells over CD8 cells infiltrating the pancreas as well as the presence of plasma cells, regulatory T cells and macrophages [13,29].

To the best of our knowledge, this is the first experimental hint for a potential involvement of memory T cells in the pathogenesis of AIP. Apart from AIP, however, memory T cells (and here in particular self-antigen-reactive CD4^+^ effector memory T cells) have been suggested to drive the progression of autoimmune diseases because of their ready effector functionality and relative longevity [35]. Along these lines, persistent antigen increases the pool of effector memory T cells, which may in turn trigger the progression of the autoimmune disease through the potent production of inflammatory cytokines, such as interferon-γ [36,37]. Indeed, we have previously observed high mRNA levels of this cytokine (as well as of interleukin-2 and interleukin-6) in pancreatic tissue of MRL/Mp mice with advanced AIP and also shown that injections of interferon-γ accelerate and aggravate the disease [12, 29].

Of course, immune cell phenotypes that fulfilled only one criterion (existence of overlapping QTLs with AIP *or* correlation with the appearance of AIP) may nevertheless be important in the progression of the disease, and their QTLs demand further investigation let alone as a controlling factor in immunity. Thus, activated CD4^+^ and CD8^+^ lymphocytes, Th17 cells and Tregs all have already been implicated in the pathogenesis of AIP [reviewed in 1]. Moreover, employing MRL/MpJ mice we have recently shown (based on immunohistochemical studies and a flow cytometric analysis of splenocytes) that the immunosuppressant drug rapamycin significantly reduces pancreatic damage by expanding Tregs of the phenotype CTLA4^+^/CD4^+^/FoxP3^+^ and a subsequent reduction of the effector T cell response [13]. We consider the fact that this part of our observations is in line with previous investigations also as supportive for our novel concept regarding the role of memory T cells in the development of murine AIP.

The overlapping QTL region for AIP and CD4^+^/CD44^high^ lymphocytes, which is located on chromosome 2 [Table 6], still spans roughly 11 Mb and contains more than 70 genes. Noteworthy, the interval contains the genes *Sjogren syndrome antigen B* (S*sb*) and *Ubiquitin protein ligase E3 component n-recognin 3* (*Ubr3*), which we have previously described as part of the QTL AIP1 [26]. The encoded proteins might be disease-relevant autoantigens and therefore deserve special attention in future studies. A full list of genes in the overlapping QTL region is provided in the S3 Table.

**Fig 4:**
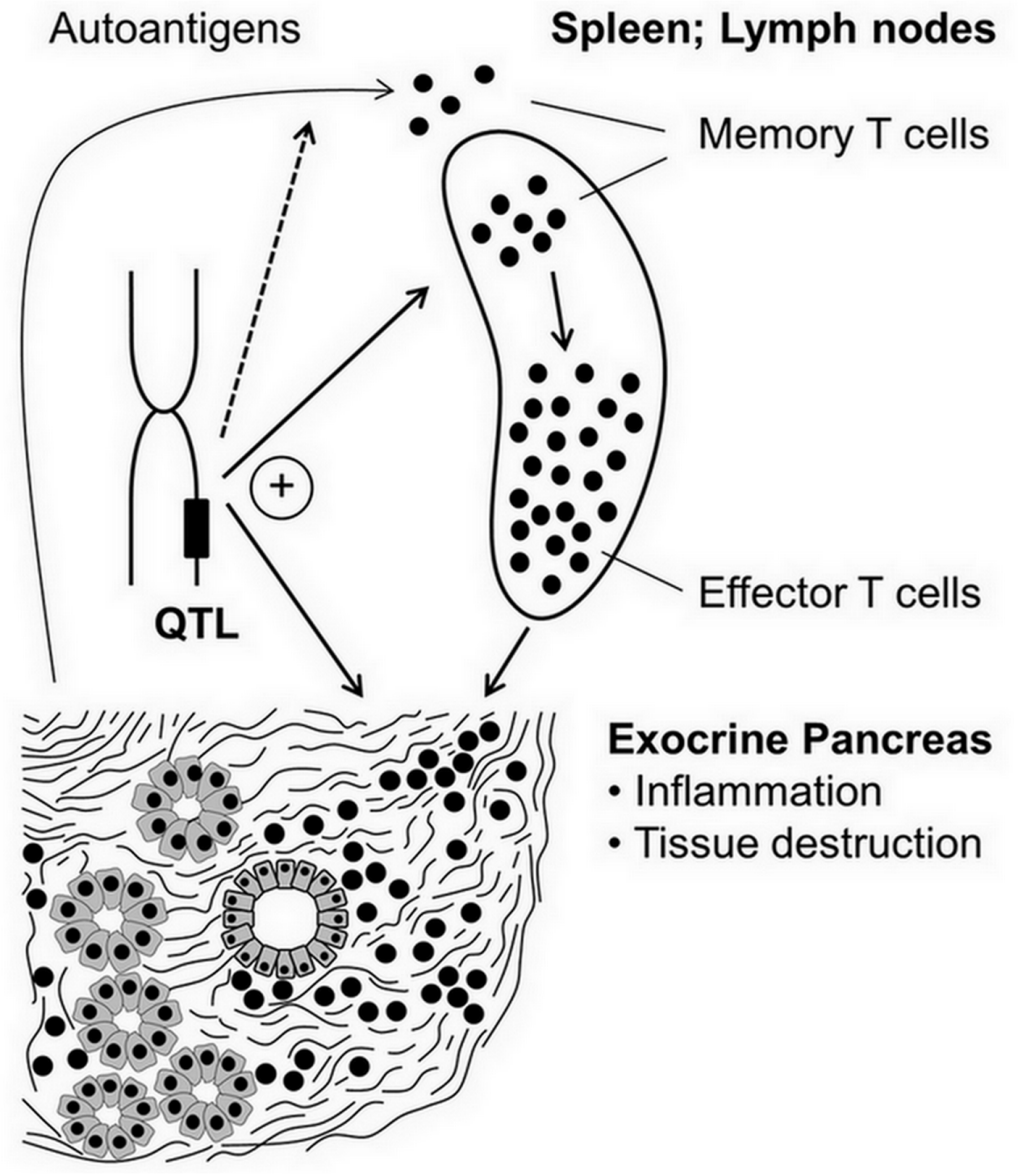
Proposed involvement of memory T cells in the pathogenesis of AIP. Autoantigens from acinar cells and pancreatic duct epithelium trigger activation of T cells, which may differentiate into T effector cells or memory T cells. The first population mediates destruction of pancreatic tissue, whereas the latter type of T cells perpetuates and enhances autoimmune reactions upon antigen re-exposure. Relative frequencies of splenic leukocyte subsets, development of AIP (pancreatic damage) and possibly expression/processing of autoantigens are controlled by QTLs. Overlapping QTLs for certain immune cell phenotypes and AIP suggest the existence of pathogenetic links.

Taken together, we hypothesize that CD4^+^/CD44^high^ memory T cells play a previously unrecognized role in murine AIP, and suggest that these cells are an important link between genetic susceptibility and development of the disease (Fig 4). Currently, fine-mapping studies are underway to narrow down AIP loci and overlapping QTLs for immune cell phenotypes, and to identify candidate genes that control the respective phenotypes.

## Supporting Information

S1 Fig FACS dot plot panels for splenocyte populations. (PPTX)

S1 Table Specific antibodies, their manufacturers and concentrations used for flow cytometry. (XLSX)

S2 Table Quantitative trait loci for splenocyte subpopulations. (XLSX)

S3 Table List of all genes in the overlapping regions of QTL AIP s1 and the QTL for CD4^+^/CD44^high^ cells on chromosome 2. (XLSX)
