# Supplementary material for: Quantitative Trait Locus Analysis Implicates CD4+/CD44high Memory T Cells in the Pathogenesis of Murine Autoimmune Pancreatitis
Source: PLoS One. 2015 Sep 1;10(9):e0136298. doi: 10.1371/journal.pone.0136298 (PMC4556487; doi:10.1371/journal.pone.0136298)
Supplement: S1 Fig — (PPTX) [file pone.0136298.s001.pptx]

## Slide 1
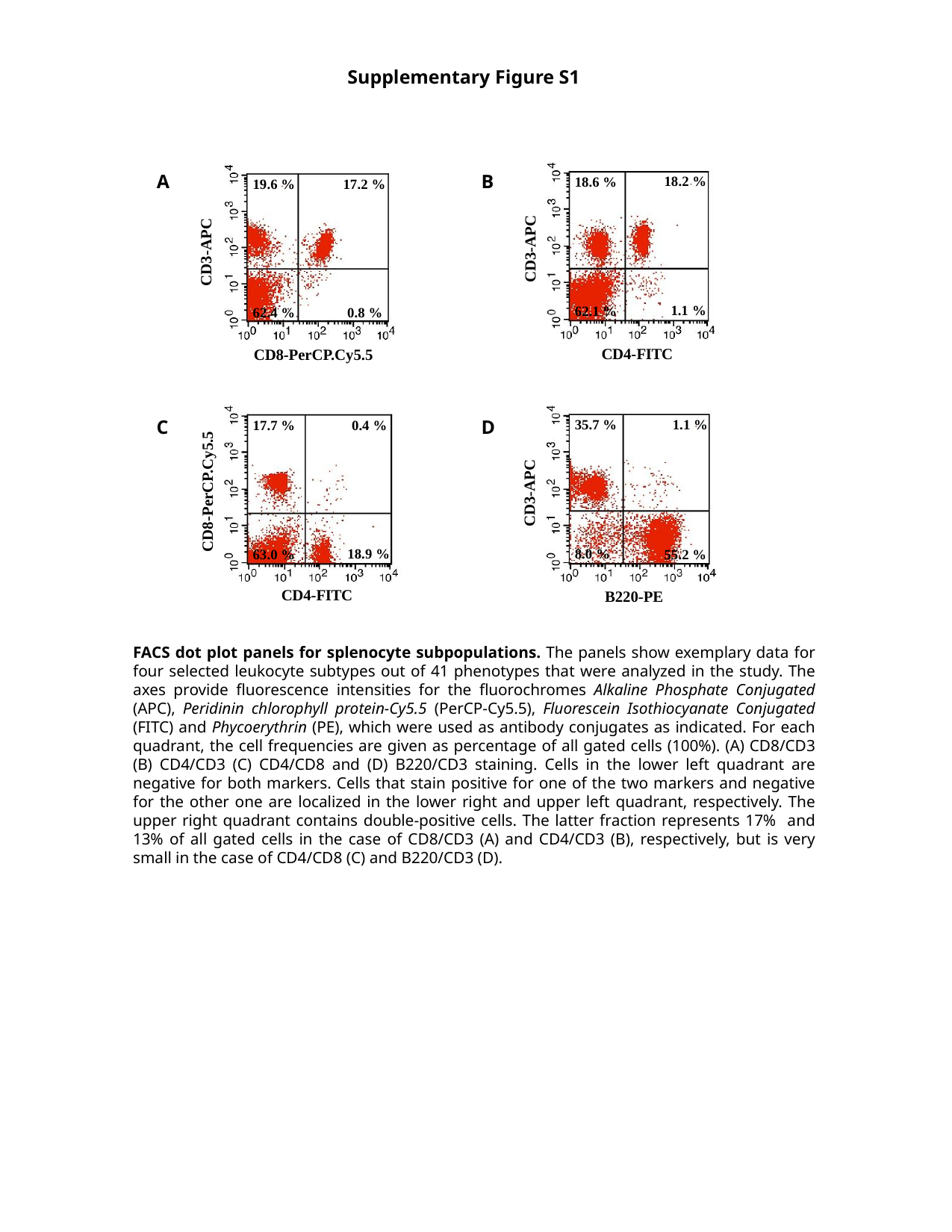

Supplementary Figure S1
A
B
18.2 %
18.6 %
17.2 %
19.6 %
CD3-APC
CD3-APC
1.1 %
62.1 %
62.4 %
0.8 %
CD8-PerCP.Cy5.5
CD4-FITC
1.1 %
35.7 %
C
D
17.7 %
0.4 %
CD8-PerCP.Cy5.5
CD3-APC
18.9 %
8.0 %
63.0 %
55.2 %
CD4-FITC
B220-PE
FACS dot plot panels for splenocyte subpopulations. The panels show exemplary data for four selected leukocyte subtypes out of 41 phenotypes that were analyzed in the study. The axes provide fluorescence intensities for the fluorochromes Alkaline Phosphate Conjugated (APC), Peridinin chlorophyll protein-Cy5.5 (PerCP-Cy5.5), Fluorescein Isothiocyanate Conjugated (FITC) and Phycoerythrin (PE), which were used as antibody conjugates as indicated. For each quadrant, the cell frequencies are given as percentage of all gated cells (100%). (A) CD8/CD3 (B) CD4/CD3 (C) CD4/CD8 and (D) B220/CD3 staining. Cells in the lower left quadrant are negative for both markers. Cells that stain positive for one of the two markers and negative for the other one are localized in the lower right and upper left quadrant, respectively. The upper right quadrant contains double-positive cells. The latter fraction represents 17% and 13% of all gated cells in the case of CD8/CD3 (A) and CD4/CD3 (B), respectively, but is very small in the case of CD4/CD8 (C) and B220/CD3 (D).
